# Supplementary material for: Brevisulcenals-A1 and A2, Sulfate Esters of Brevisulcenals, Isolated from the Red Tide Dinoflagellate Karenia brevisulcata
Source: Toxins (Basel). 2021 Jan 22;13(2):82. doi: 10.3390/toxins13020082 (PMC7911007; doi:10.3390/toxins13020082)
Supplement: Supplementary file 1 [file toxins-13-00082-s001.pdf]

Supplemental Material: Brevisulcenals-A1 and A2, Sulfate Esters of Brevisulcenals, Isolated from the Red Tide Dinoflagellate *Karenia brevisulcata*

Masayuki Satake, Raku Irie, Patrick T. Holland, D Tim Harwood, Feng Shi, Yoshiyuki Itoh, Fumiaki Hayashi and Huiping Zhang

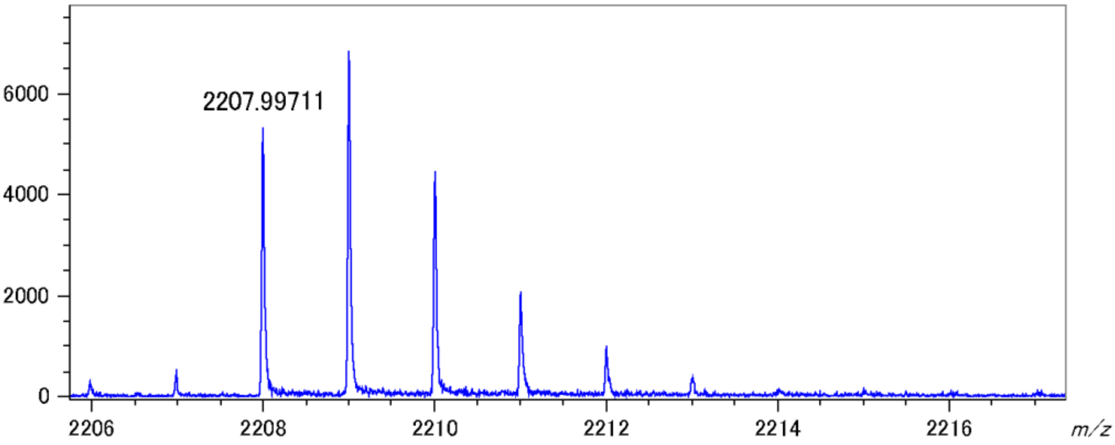

Figure S1. Spiral MALDI MS of KBT-A2.

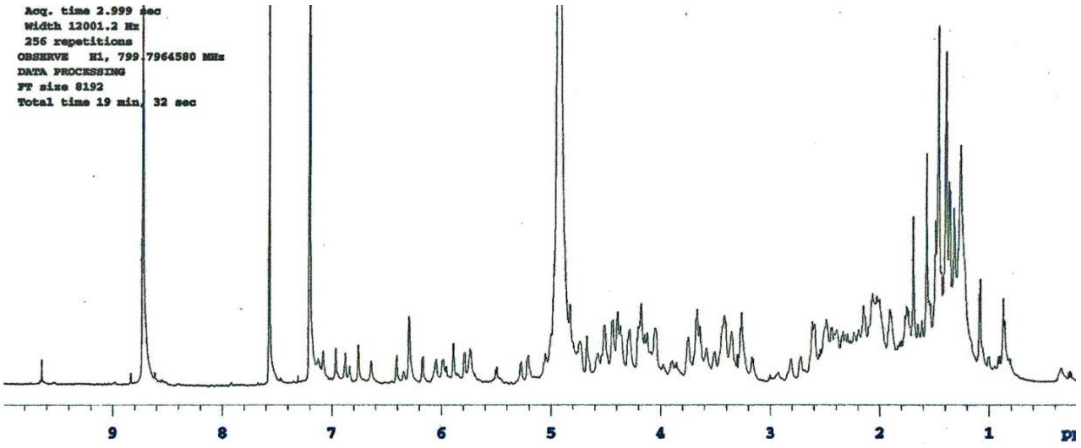

Figure S2. ^{1}H NMR spectrum of KBT-A2 (800 MHz, pyridine-d_5).

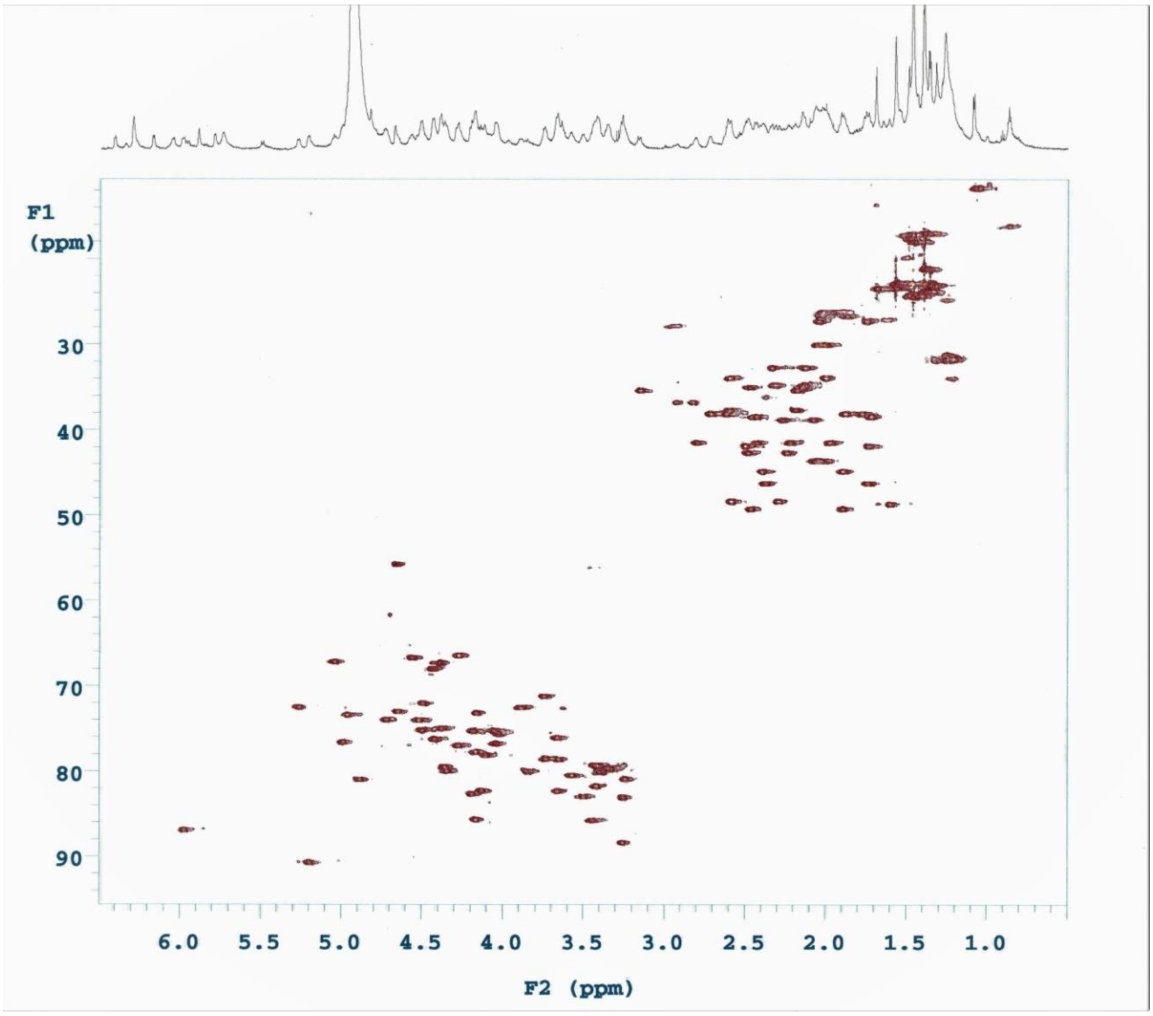

Figure S3. HSQC of KBT-A2 (800 MHz, pyridine-d_5).

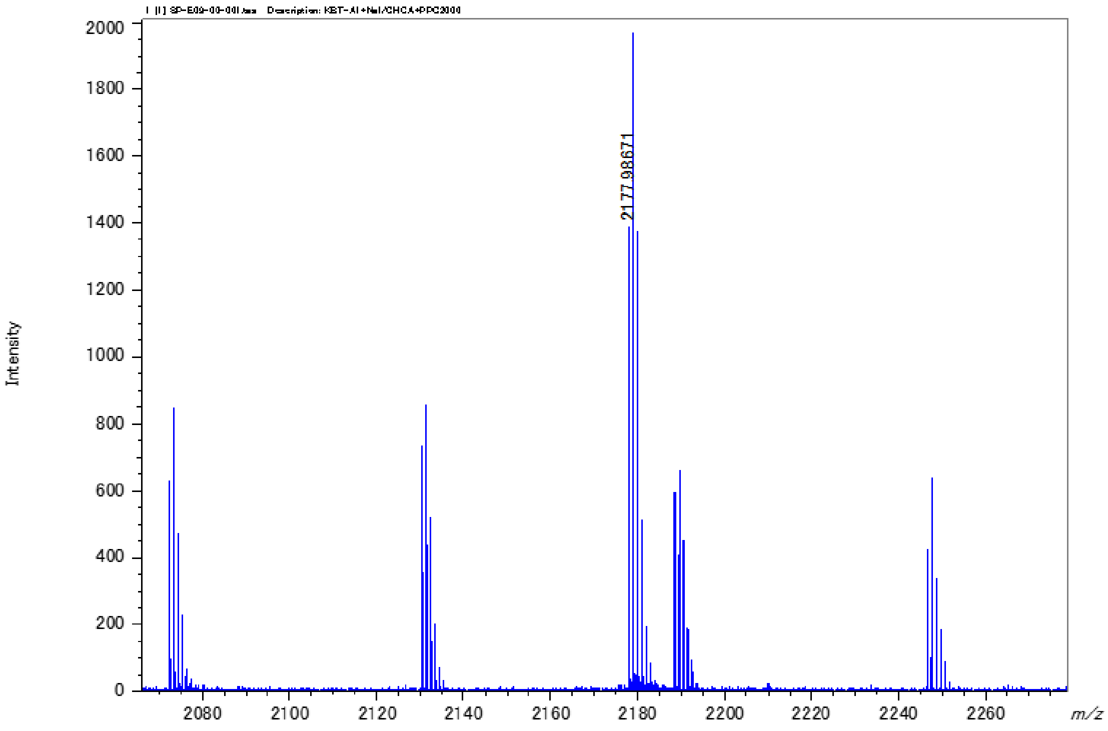

Figure S4. Spiral MALDI MS of KBT-A1.

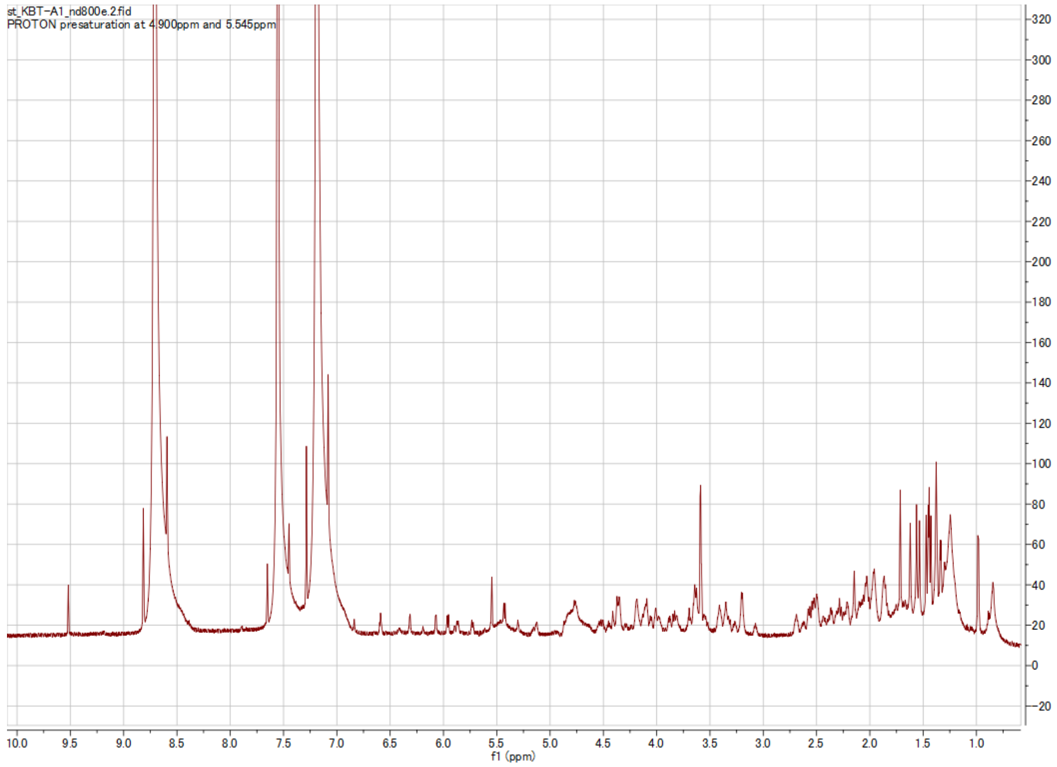

Figure S5. ^{1}H NMR spectrum of KBT-A1 (800 MHz, pyridine-d_5).

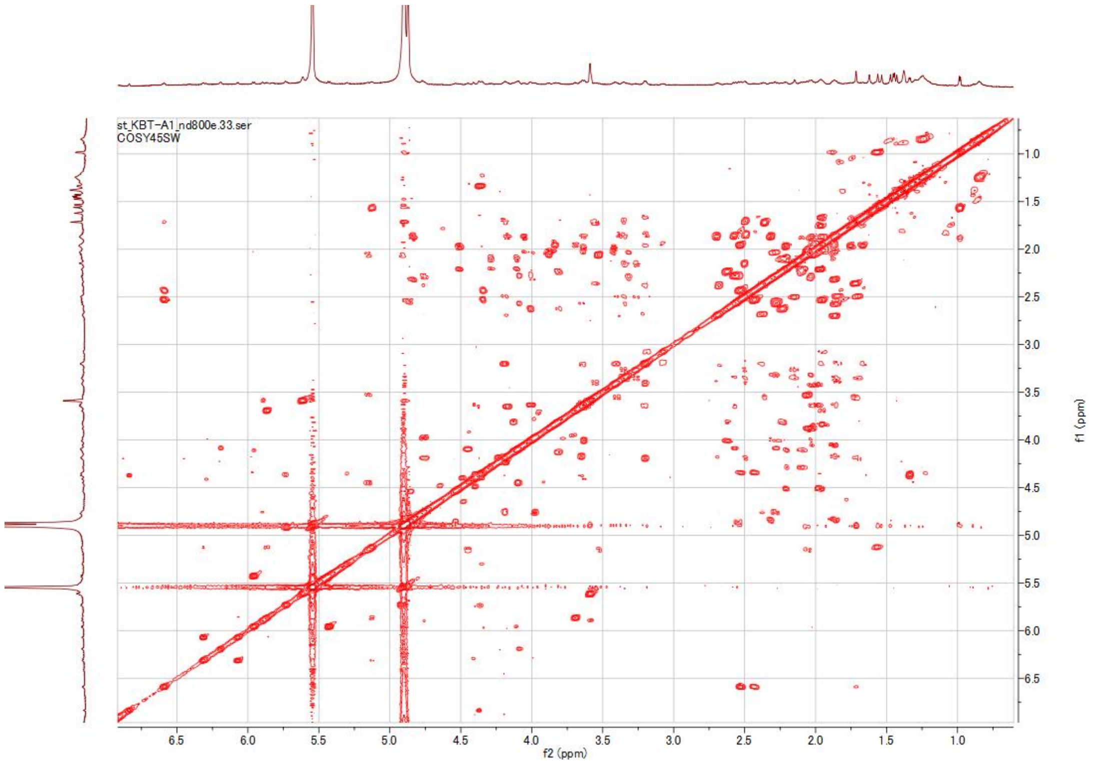

Figure S6. ^1H-^1H COSY of KBT-A1 (800 MHz, pyridine-*d*$_5$).

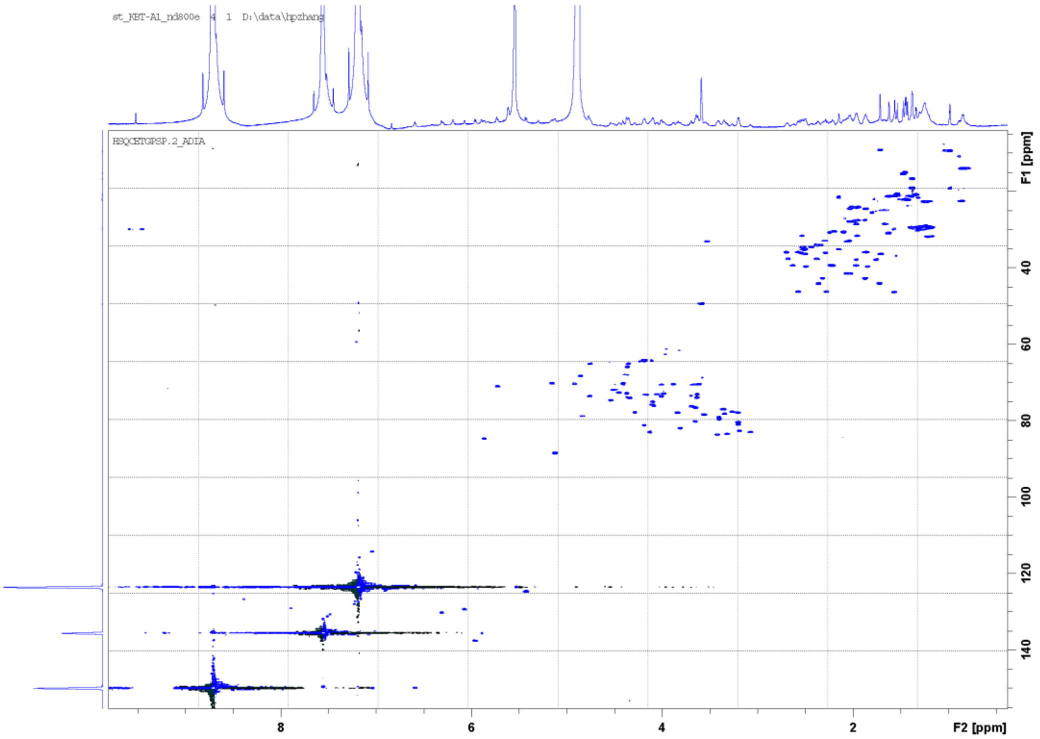

Figure S7. HSQC of KBT-A1 (800 MHz, pyridine-*d*$_5$).

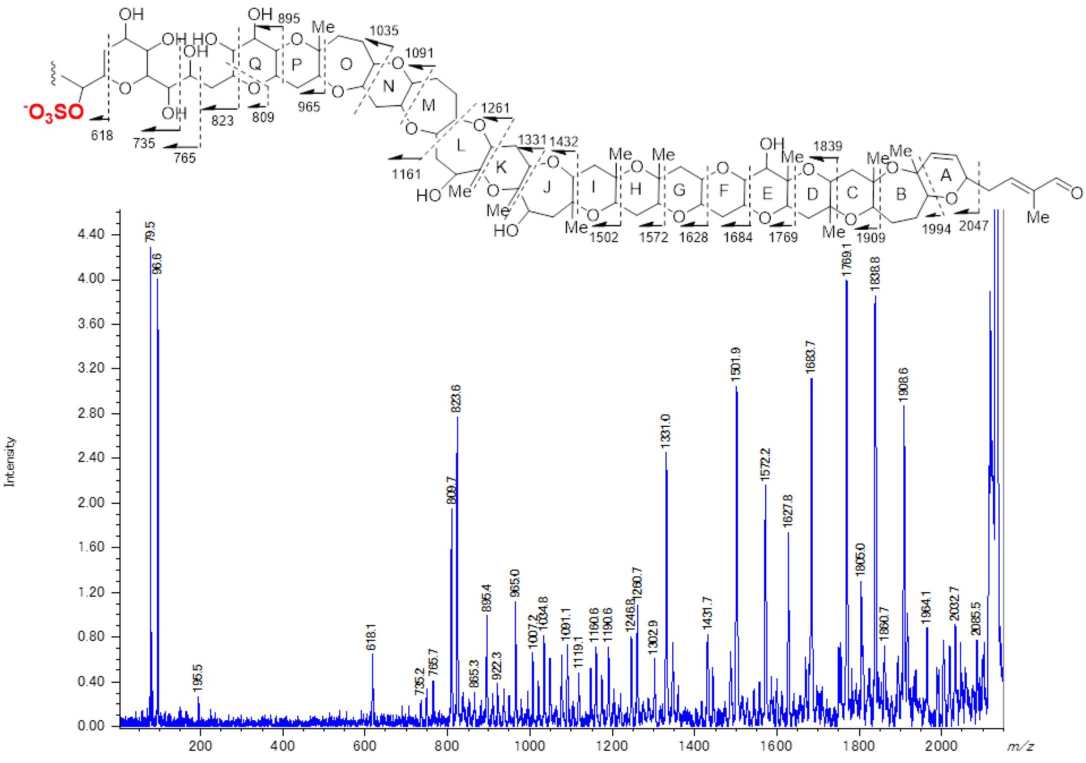

Figure S8. Spiral MLADI TOF-TOF of KBT-A1.

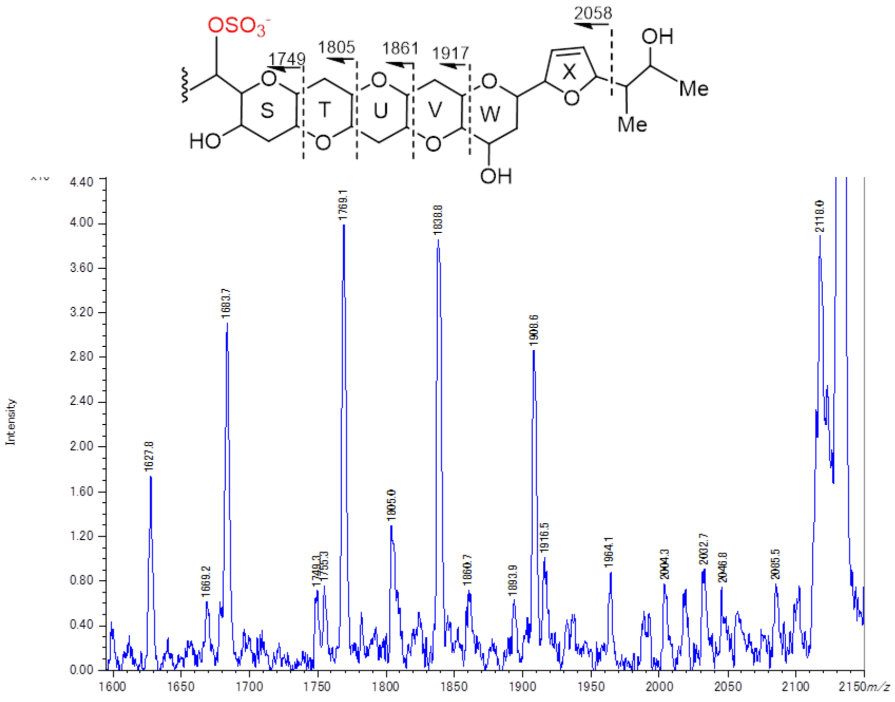

Figure S9. Spiral MALDI TOF-TOF of KBT-A1 (*m/z* 1600–2150).
